# Brain fog in Parkinson’s disease: unraveling mechanisms and measuring impact

**DOI:** 10.3389/fneur.2025.1571079

**Published:** 2026-01-07

**Authors:** Timothy R. Elliott, Xavier Valencia, Susy Chen, Andre Matta

**Affiliations:** 1 Department of Educational Psychology, Texas A&M University (Emeritus), College Station, TX, United States; 2 Ventus Therapeutics U.S., Inc., Waltham, MA, United States

**Keywords:** brain fog, Parkinson’s disease, neuroinflammation, dopamine, neuroimmunomodulation, fatigue, cognitive dysfunction, Fatigue and Altered Cognition Scale

## Abstract

Inconsistent colloquial and professional uses of the term “brain fog” have undermined the potential for empirical study of this symptom complex, and there is no consensus about how to diagnose or treat it. Yet brain fog is frequently reported by patients with post-acute sequelae of SARS-CoV-2 (PASC) and other chronic conditions, often presenting as cognitive difficulties such as memory issues and attention deficits, accompanied by central fatigue and sometimes depression. This symptom complex is also common in Parkinson’s disease (PD). One objective of this article is to propose a theoretical model that conceptualizes brain fog in PD in terms that can guide its measurement and treatment. Neuroinflammation, dopaminergic dysfunction, and immune system interactions are examined as potential mechanisms. We present the Fatigue and Altered Cognition Scale (FACs), a patient-report tool designed to assess brain fog in clinical and research settings that has been validated in traumatic brain injury and PASC. Proper diagnosis and monitoring of brain fog are important because early research suggests that existing medications, such as methylphenidate, and natural substances, such as carnosic acid, have the potential to alleviate its symptoms. Ongoing research is crucial to establish a clear definition of brain fog and identify effective treatments in PD and other neurodegenerative disorders.

## Introduction

Co-occurring complaints of fatigue and brain fog represent a debilitating symptom complex that has a strikingly similar manifestation in more than a dozen chronic conditions, such as traumatic brain injury (TBI), multiple sclerosis (MS), chronic fatigue syndrome, fibromyalgia, lupus, Sjögren’s syndrome, postural orthostatic tachycardia syndrome, hypoparathyroidism, and celiac disease (1, 2). The frequency of this symptom constellation across many chronic health conditions suggests it may be a transdiagnostic syndrome (3, 4) and a potential public health concern (5). Until recently, however, brain fog was poorly investigated, and the inconsistent, if not circular, colloquial and professional uses of the term undermine an operationalization of the construct required for empirical study. At present, there is no consensus about how to diagnose or treat brain fog.

Brain fog is reported by 25 to 90% of patients who develop post-acute sequelae of SARS-CoV-2 infection (PASC) (6). Central fatigue is also a principal symptom of PASC (1, 7). In comparison to peripheral fatigue—muscular impairment or exhaustion due to exertion—central fatigue, also called mental fatigue, is a subjective report of difficulty initiating and maintaining activity and attending to tasks that require sustained mental effort (8). The very high prevalence of brain fog in PASC has prompted a flurry of research, and there is new evidence that brain fog is a central nervous system dysfunction that has cardinal symptoms, separate from peripheral fatigue and depression, and has the potential to be measured objectively.

In this commentary, after discussing characteristics of brain fog identified in PASC, we focus on brain fog in Parkinson’s disease (PD). We propose an empirical model that may explain why brain fog develops in PD and suggest the use of a previously published scale for measuring brain fog clinically and in clinical trials.

## Brain fog in PASC

To tease out more exactly what deficits constitute brain fog, a research team in Spain conducted comprehensive neuropsychological testing of 170 patients with PASC who had cognitive complaints (2). The results showed brain fog is associated with objective impairment in attention and episodic memory and is correlated with but separate from central fatigue. The effect of depression in brain fog was indirect and mediated through central fatigue.

A study in Ireland included 71 patients with PASC who self-reported brain fog (yes/no) (9). Key indicators of brain fog included word-finding difficulties, memory impairment, and reduced cognitive response times. Others were dizziness, myalgia, reduced grip strength, reduced gait speed, and poorer scores on the Chalder Fatigue Scale, which measures both peripheral and central fatigue. These neurologic and psychomotor consequences make it easy to understand why patients affected by PASC-related fog report substantially diminished quality of life (QoL) (9–11).

Intriguingly, small studies suggest brain fog may be a measurable phenomenon in PASC. Patients have exhibited lower functional connectivity in multiple brain regions on magnetic resonance imaging (12), hypometabolic regions of the cingulate cortex on cerebral fluorodeoxyglucose positron emission tomography (FDG-PET) (13), and changes in their quantitative electroencephalography profile compared with profiles recorded before they developed PASC (14).

## Brain fog in PD

Although empirical studies of brain fog associated with PD are lacking, qualitative research reveals that patients living with PD and their families report problems they term “brain fog” (15, 16). The lack of consensus in defining brain fog in PD, however, is on full display in recent studies of verbatim responses to open-ended questions in a large-scale survey sponsored by the Michael J. Fox Foundation. In a study of more than 25,000 respondents, a machine learning algorithm included the term “brain fog” in a domain labeled “mental alertness/awareness” rather than domains of “memory,” “concentration/attention,” and “cognitive slowing” (17). In another study of the same survey (*n* > 21,000), the same algorithm excluded “brain fog” from the “mental alertness/awareness” domain and categorized it under “cognitive impairment not otherwise specified” (18). In both studies, the most bothersome problems were with memory and concentration/attention.

Consistent with our understanding of brain fog in other chronic conditions, patients with PD are often said to experience “confusion” (19) and cognitive impairment in PD characterized by attentional disturbances (20). The most relevant research about brain fog in PD concerns subjective cognitive decline (SCD)—subjective cognitive complaints without concurrent objective cognitive deficits as measured by validated cognitive scales. When 139 patients with newly diagnosed PD underwent comprehensive neuropsychological evaluation, the prevalence of SCD was 28% (21). As in the Spanish study of brain fog in PASC (2), the most commonly affected domains were memory (28% of patients with SCD) and attention/working memory (26%). Overlapping symptoms in multivariable linear regression analysis were anxiety and depression.

Italian researchers asked 90 consecutively enrolled non-demented patients with PD to complete the Parkinson’s Disease Cognitive Functional Rating Scale (subjective measure) and the Montreal Cognitive Assessment (MoCA, objective measure) (22). Twenty-nine patients (32%) were classified as “underestimators” who had SCD but no objective cognitive impairment. Scores on the Fatigue Severity Scale and the Beck Depression Inventory distinguished underestimators from accurate estimators and overestimators (those with objective impairment but no self-reported complaints).

Using FDG-PET, a German team documented neural correlates of SCD in PD (23). Among 18 PD patients determined to have SCD, greater concern about cognition in everyday situations correlated with greater hypometabolism in middle frontal, middle temporal, and occipital areas of the brain, as well as the angular gyrus.

As it does in PASC, brain fog in PD can have profound negative effects on QoL. In an online assessment of 612 individuals with PD, confusion more than quadrupled the odds of poor QoL (McGill Quality of Life Questionnaire), and memory loss more than tripled it (24). In a similar study of 139 patients, confusion and memory problems were significantly correlated with poor QoL reported on the Parkinson’s Disease Questionnaire–39 (25).

## Toward an empirical model of brain fog in PD

Recent attempts to advance our understanding of possible mechanisms that may account for the transdiagnostic nature of co-occurring brain fog and fatigue have focused on issues of energy required for cognitive processing (26) and the neural pathways involved in cognitive and motivational control (27). Ideally, clinicians assisting patients with PD will benefit from a theoretical model that conceptualizes brain fog in terms that can guide its measurement and treatment. One such model conceptualizes brain fog as a symptom of pituitary dysfunction that can occur in the wake of TBI and is usually accompanied by central fatigue (28). Reasoning from this model, research has examined the ability of growth hormone replacement therapy to alleviate these symptoms (29–31). Clinical case studies of patients with TBI (31) and PASC (32) have been promising, but randomized clinical trials are needed.

As a transdiagnostic entity, brain fog may have different causes, but in PASC and MS, neuroinflammation clearly has a key role. Fernández-Castanẽda and colleagues recently reported similarities between brain fog in PASC and “chemo fog,” or cancer therapy–related cognitive impairment (CRCI) (33, 34). During chemotherapy or radiation, elevations in neurotoxic cytokines and reactive microglia can lead to cascades of multicellular events that negatively affect gray and white matter plasticity, which is important for cognition. Reminiscent of CRCI, the researchers found elevated reactivity of microglia and macrophages in subcortical and hippocampal white matter in mice following mild respiratory COVID-19, and the elevation persisted 7 weeks post-infection. In murine cerebrospinal fluid, cytokine levels remained persistently elevated, including levels of CCL11, which has been causally linked to cognitive impairment in normal aging (35). Moreover, CCL11 was elevated in plasma from patients with PASC—but only those with cognitive symptoms.

A case–control study pointed to a different impact of neuroinflammation in PASC (36). Radiolabeled PET showed that, compared with healthy controls, patients with PASC who had depressive symptoms and/or cognitive impairment had higher levels of the translocator protein, a marker of microglial activation, in cortex, hippocampus, and other brain regions. Very similar results were reported from a study of 28 patients with MS, and the level of the translator protein in white matter and cortex correlated with decreased information processing speed and neurologic disability (37).

One intriguing question is whether the proinflammatory process launched by hyperactivated microglia is mediated by the nod-like receptor protein-3 (NLRP3) inflammasome. Activation of NLRP3 in monocytes and microglial cells by alpha-synuclein is well documented in PD, and it can be triggered by dopaminergic degeneration even in the absence of alpha-synuclein aggregates (38–40). Serum levels of NLRP3 and interleukin-1β, a potent proinflammatory cytokine, are elevated in PD patients, and there is a linear correlation of NLRP3 with *α*-synuclein (39).

MS is a good model for us to understand the impact of neuroinflammation and dopaminergic dysfunction on mental processes in other neurodegenerative diseases such as PD. We hypothesize that lessons learned from MS could be applicable to PD as the two diseases share some common pathophysiological aspects, such as microglial-driven neuroinflammation and dopaminergic dysfunction. Indeed, neuroimmune interaction is considered one of the most promising directions in MS research. Dopamine is a direct mediator of interactions between the immune and nervous systems and can influence the course of MS by modulating immune cell activity and cytokine production (41–43). Fatigue, the most common symptom in MS (44), has been attributed to a dopamine imbalance that disrupts communication between the striatum and prefrontal cortex (45). Many patients who receive disease-modifying treatment of MS, most of them with potent anti-inflammatory effects, report improvements in fatigue, cognition, or both (46–51). Amantadine, which is used off-label to treat fatigue in MS, has also improved cognitive function in some studies (52, 53). Amantadine has multiple mechanisms of action, but its effect on brain fog may be attributable to its interference with dopamine transmission.

We postulate that, as in MS, dopaminergic dysfunction is a mechanism of brain fog in PD. Neurons are designed to live and function in a peaceful sanctuary, thanks to the blood–brain barrier and the work of glial cells in maintaining brain homeostasis and regulating inflammatory responses. Any disturbance in the surrounding microenvironment may lead to neuronal dysfunction. PD constitutes a situation where dopaminergic neurons are not only scarce (some already died) but also under attack from inflammation, alpha-synuclein accumulation, tau phosphorylation, and so forth. This imbalance could lead to a constellation of symptoms, including objective cognitive decline, mood disorders, and brain fog.

Other neurotransmitter pathways, such as cholinergic and serotoninergic networks, do not seem to play a role in brain fog. Deterioration in cholinergic projections from the basal nucleus of Meynert to the cortex is one hallmark of cognitive dysfunction in Alzheimer’s disease (AD), and acetylcholinesterase inhibitors produce modest, observable improvement in the cognitive symptoms of AD. But the evidence for anticholinesterase inhibitors in PD is not as clear as in AD (54). Neither does brain fog seem to be associated with serotonergic dysfunction, as selective serotonin reuptake inhibitors can fail to completely relieve brain fog–like symptoms in patients with depression (55, 56).

Finally, there are confounding factors in studying the mechanism of brain fog in PD and in diagnosing and treating it. Cognitive impairment in PD is often associated with depression or adverse effects from levodopa, dopamine agonists, antipsychotics, or antidepressants (57–60). Clinicians should keep in mind that self-report measures of depression include items that overlap with symptoms of brain fog and fatigue (e.g., tiredness, concentration difficulties, psychomotor retardation): Total scores from self-report measures do not indicate a depressive disorder. Brain fog and fatigue can exist in the absence of depressed mood and anhedonia.

## Future directions

Early detection of brain fog and altered cognition is critical for clinical management strategies that might delay PD dementia. To date, much research into brain fog has relied on time-consuming neuropsychological testing, which is too cumbersome and impractical for routine use in clinical encounters and monitoring symptom response to treatment, and may be compromised by patient fatigue. For assessment of fatigue, at least 14 scales are available to measure PD patients’ self-reports, but most lack a consistent definition of fatigue (61). Furthermore, many of them, such as the Parkinson Fatigue Scale (62), emphasize the physical aspects of fatigue rather than central fatigue. Indeed, Chen and colleagues determined in a systematic review that no single scale is sufficient for diagnosing central fatigue in PD (61).

To address these problems, Elliott et al. (63) developed the Fatigue and Altered Cognition Scale (FACs) to measure the presence and severity of brain fog and central fatigue over the past 2 weeks (Table 1). The format expedites patient reporting and clinician use across platforms, including mobile phones and tablet and laptop computers. The FACs two-factor structure and internal consistency (*α*’s ranging from 0.91 to 0.97) has been validated in TBI (64), MS (65), and PASC (6), where no FACs item significantly overlapped with the cardinal symptoms of depression (sad mood, loss of pleasure) or anxiety (excessive worry). Construct validity of the two factors has also been established with measures of physical and cognitive fatigue, loss of vitality, attention deficits, and subjective cognitive complaints (65), as well as measures of cognitive and emotional burnout (66). Other scales have been recently developed to measure brain fog alone (67–69), although only one of these was developed and validated with a clinical sample (67). Evidence about their utility in specific chronic health conditions is lacking.

**Table 1:** The Fatigue and Altered Cognition Scale.

Item no.	Item text
*Fatigue scale*	
1	I felt fatigued
2	I felt alert^*^
6	I felt worn out
7	I felt sluggish
8	I felt run down
10	I had the energy to do what I wanted to do^*^
13	I had to force myself to get things done
15	I felt tired
17	I had to struggle to finish what I started to do
20	I had problems feeling energetic no matter if I slept or napped
*Altered cognition scale*	
3	I lost track of what I was going to say
4	I was forgetful
5	I had trouble concentrating
9	I had trouble focusing on things I wanted to do
11	I was easily confused
12	I felt “spaced out” like I was in a fog
14	I was clear-headed^*^
16	I did not process things as quickly or accurately as I should have
18	I had trouble paying attention
19	It was hard for me to make up my mind and reach a decision

^*^Reverse coded before conducting item analyses.

Modified from this table in Elliott et al. (63) with permission under the auspices of a Creative Commons Attribution License.

The FACs was designed to expedite clinical assessment and treatment of co-occurring brain fog and central fatigue. Its use should complement current practice. The Montreal Cognitive Assessment (MoCA) is internationally recommended to screen for mild cognitive impairment among PD patients (70, 71). It is an objective measure that informs case conceptualization and initial treatment plans including the probable need for a full neuropsychological battery to determine specific deficits (70, 72). The FACs can be used in case conceptualization, but it is purpose-built for serial administrations in the clinic to assess a patient’s subjective experience of symptoms over time in response to treatment, guiding clinical decisions to alleviate patient suffering and complaints. The FACs has features that meet several criteria for “pragmatic validity” (73). It is brief and it can be self-administered with minimal respondent burden, sensitive to change, and it is designed to provide information that guides therapeutic decisions and facilitates patient-clinician communication about issues important to the patient.

Now that an efficient measurement tool is available, brain fog should be an endpoint in neurologic clinical trials. In addition, the FACs could be used to plan and monitor treatment of brain fog. For example, preliminary research shows that methylphenidate, a dopaminergic psychostimulant, is associated with subjective improvement in memory and concentration in PASC (74) and alleviates fatigue in individuals with MS (44) and PD (75). The FACs could help document objective findings in further research. The validity and reliability of the FACs in PD warrants future study.

As another example, carnosic acid, a derivative of the herb rosemary, has been shown to exert neuroprotective effects in multiple *in vivo* models of PD and AD (76). Its mechanism in this regard is phase 2 enzyme induction initiated by activation of the KEAP1/NRF2 transcriptional pathway, which in turn attenuates NLRP3-driven inflammation, including in human macrophages (76, 77). Carnosic acid is hypothesized to have application as a therapy for brain fog in PASC, AD, and PD (77), but at this writing no clinical study has been published.

This progress is encouraging, but the research into brain fog has been inconsistent, which obstructs a clear understanding of the phenomenon. The field will benefit from continued work to develop a working definition of brain fog, establish the mechanisms that drive it, and determine therapeutic targets. Further, it is apparent that cultural and language differences in symptom reports merit empirical scrutiny. A recent translation study of the FACs into European Portuguese found 12 items had terms or phrases that required cultural substitutions to ensure the items were understandable and meaningful (66). Programmatic and systematic research should empirically advance our understanding and help us rule out faulty and ineffective approaches.

## Data Availability

The original contributions presented in the study are included in the article/supplementary material, further inquiries can be directed to the corresponding author.
